# The Preferred Odor Characteristics of Cooked Medium-Milled Fragrant Simiao Rice

**DOI:** 10.3390/foods15020356

**Published:** 2026-01-19

**Authors:** Rui Lai, Jie Liu, Qing Huang, Xiaoji Fei, Hongzhou An, Qian Lin, Yanru Li

**Affiliations:** 1 College of Food Science and Engineering, Henan University of Technology, Zhengzhou 450001, China; lairuilr@163.com (R.L.); huangqing_qing@163.com (Q.H.); feixiaoji7912@163.com (X.F.); 2 National Engineering Research Center of Wheat and Corn Further Processing, Zhengzhou 450001, China; 3 R & D Innovation Office, Guangzhou Lingnan Suiliang Grain Co., Ltd., Guangzhou 510000, China; linqian_guwu@126.com (Q.L.); liyanru1004@gmail.com (Y.L.)

**Keywords:** medium-milled fragrant Simiao rice, GC–MS, odor activity values, volatile compound, discriminating volatiles

## Abstract

Medium-milled rice is increasingly valued for its health benefits and distinctive aroma, which differs from that of white rice because differences in milling degree modify the content of lipids and other aroma precursors. However, its aroma profile remains underexplored. This study aimed to systematically analyze aroma differences among four Simiao rice cultivars after medium milling (8% degree of milling) and to elucidate the chemical basis underlying consumer preference. Odor sensory evaluation identified Xiangzhuxiang as the cultivar with the highest aroma acceptance. Subsequently, gas chromatography–olfactometry–mass spectrometry and odor activity value analysis characterized the volatile profile, identifying 45 volatile compounds across the four cultivars, including 17 key odor-active components. Multivariate statistical analysis pinpointed the discriminating key odor-active compounds responsible for the superior aroma quality of Xiangzhuxiang. The results showed that (*E*,*E*)-2,4-decadienal and indole (VIP > 1.0, FDR-adjusted *q* < 0.05, FC > 1.2, OAV > 1.0, confirmed by GC-O) significantly increased the aroma scores of Xiangzhuxiang; imparted nutty, fatty, and sweet notes; and thus played a decisive role in shaping its characteristic aroma. Moreover, the moderate levels of hexanal and octanal in Xiangzhuxiang facilitated its characteristic aroma expression. These findings provide a basis for developing premium fragrant Simiao rice cultivars optimized for medium milling.

## 1. Introduction

Rice (*Oryza sativa* L.) is a vital staple food, particularly in Asia, where over half of the global population resides [1]. Rice can be classified into two aroma-based categories: fragrant and non-fragrant [2]. Fragrant rice is generally more preferred by consumers and costs more in the market [3]. China, the largest producer and consumer of rice globally, encompasses six agro-climatic zones specifically dedicated to rice cultivation [4]. Fragrant Simiao rice is one of the prime examples of fragrant rice in Southern China, esteemed for its robust aroma and delightful taste.

Aroma is a significant determinant of rice quality, serving as a key criterion for consumers when accepting and evaluating rice, particularly fragrant varieties [3]. Research shows that rice odor is a highly heritable trait [5]; thus, diverse rice cultivars exhibit distinct volatile profiles. Multiple prior research endeavors have documented variations in aroma among different rice cultivars, such as Basmati [6], Suxiang [5], Khao Dawk Mali 105 [7], etc. The focus of these related studies was primarily on cooked or raw well-milled rice, with a limited number of studies also examining cooked brown rice. Recent research has shown that retaining a higher percentage of the bran layer in milled rice could increase grain yields, energy conservation, and enhance human health benefits [8]. Due to the increasing health consciousness among consumers, there has been a growing preference and demand for medium-milled rice [9,10]. Regrettably, the existing research in this area remains scarce.

Recent research has demonstrated that a lower degree of milling (DOM) could influence the odor of cooked rice. Billiris et al. (2012) reported that brown rice displayed significantly higher bitterness than well-milled rice, attributed to the presence of intense odors like cooked grain, nutty, burlap, wet cardboard, feedy, and woody odors; in contrast, brown rice has lower levels of sulfury flavor compared to well-milled rice [8,11]. The odor of cooked rice was primarily determined by the composition and content of volatile compounds in it, originating from the decomposition of its precursor substances, such as unsaturated fatty acids [12]. These precursor substances were highly concentrated in the bran layer of rice [5]. Previous studies have indicated that milled black rice displayed significantly reduced levels of total volatiles, specifically acids, esters, and alcohols, in comparison to its unmilled counterpart [13]. As observed by Li et al. (2023), increasing the DOM from 0% to 9% led to a decline in the content of various volatiles, such as (*E*)-2-decenal, methyl palmitate, benzoic acid, and a series of alkanes and aromatic compounds including undecane, dodecane, tetradecane, styrene, and naphthalene [5]. Similarly, Mahmud et al. (2017) found a progressive reduction in the content of benzene, hexan-3-one, glutaraldehyde, and 2-pentylfuran identified in Cheonjihyang-1-se, a fragrant rice cultivar, as the DOM increased from 1.33% to 11.0% [12]. Therefore, the variations in odor between different cultivars of well-milled rice might significantly differ from the variations in odor between their medium-milled rice. Minor variations in the aroma profile of cooked rice could significantly impact consumer preference. Hence, any modification to the DOM might alter the market acceptability of specific rice cultivars, thereby exerting a decisive impact on their competitiveness in the marketplace. Therefore, it is necessary to investigate odor variations within the medium-milled rice of different rice cultivars.

In this study, four fragrant Simiao rice cultivars widely cultivated in Southern China and possessing significant breeding potential were systematically investigated to characterize their odor profiles. Initially, odor sensory evaluation was conducted to select medium-milled rice cultivars with higher aroma scores. Subsequently, gas chromatography-mass spectrometry (GC–MS), gas chromatography-olfactometry (GC–O), and odor activity value (OAV) analyses were employed to elucidate the specific volatile components and odor-active compounds unique to the target cultivar. Finally, multivariate statistics analysis was applied to determine the discriminating key odor-active compounds responsible for cultivar-specific odor differentiation. The outcomes of this study are significant for flavor breeding of medium-milled rice.

## 2. Materials and Methods

### 2.1. Materials

Four cultivars of fragrant Simiao paddy, including 19-xiang, Meixiangzhan, Xiangyaxiangzhan, and Xiangzhuxiang, were sourced from the Guangdong Academy of Agricultural Sciences. Anhydrous sodium sulfate, anhydrous ether, and dichloromethane were sourced from Tianjin Tianli Chemical Reagent Co., Ltd. (Tianjin, China). A standard mixture of n-alkanes ranging from C_5_ to C_30_ was obtained from Agilent Technologies (Palo Alto, CA, USA). All standard odorant compounds, including the internal standard 2-methyl-3-heptanone, were acquired from Macklin Biochemical Co., Ltd. (Shanghai, China). Chromatographic-grade methanol was used, while all other reagents were of analytical grade.

### 2.2. Rice Milling

The optimal range of milling degree for each cultivar was established previously by assessing both milling quality and eating quality. Thus, to satisfy both these cultivar-specific milling degree ranges and the Chinese national standard GB/T 1354–2018 (Milled Rice) criteria for medium-milled rice (bran degree: 2–7%), degree of milling (DOM) was fixed at 8% for all samples. This standardization ensured that all cultivars were milled under identical conditions.

All medium-milled rice was obtained through husking (THU35C, Satake, Japan) and milling (TM05C, Satake, Japan) processes, with all final products exhibiting a DOM of 8%. DOM was quantified using the equation below:

$$\mathrm{DOM}\;=\;1\;-\;\frac{\mathrm{weight}\;\mathrm{of}\;\mathrm{milled}\;\mathrm{rice}\;(g)}{\mathrm{weight}\;\mathrm{of}\;\mathrm{brown}\;\mathrm{rice}\;(g)}\;\times 100\%$$

### 2.3. Rice Cooking

The preparation of cooked medium-milled rice followed the procedure specified in the Chinese national standard (GB/T 15682-2008 [14]). For each sample, 10 g of rice was transferred into a 500 mL beaker, mixed with 300 mL of ultrapure water, and gently stirred and rinsed twice within an 8 min timeframe. Ultrapure water was added to the drained rice at a ratio of 1:1.6 (*w*/*v*), and the mixture was left to soak at 25 °C for 30 min. Subsequently, the soaked rice was steamed for 40 min in an aluminum container placed above boiling water, followed by a 20 min equilibration period without heat. Once the cooking process was complete, the rice samples were cooled to ambient temperature prior to analysis. Cooked medium-milled fragrant Simiao rice from four different cultivars (19-xiang, Meixiangzhan, Xiangyaxiangzhan, and Xiangzhuxiang) were prepared accordingly and labeled as CV1, CV2, CV3, and CV4, respectively.

### 2.4. Odor Sensory Analysis

Sensory evaluation was performed in accordance with the Chinese national standard (GB/T 15682-2008). Twelve trained assessors (balanced by gender, aged 21–43 years), who were selected and trained according to ISO 8586:2023 [15], participated in the odor preference evaluation of cooked rice samples. Candidates were screened for normal olfactory acuity and completed several practice sessions using reference odorants relevant to cooked fragrant rice before the formal tests. Samples were individually coded with random numbers and presented in Petri dishes, with a 3 min break between each evaluation. To minimize cross-sample interference, participants rinsed their mouths with mineral water before proceeding to the next sample. Aroma intensity was rated according to the following scale: Strong aroma (91–100 points): Pronounced rice fragrance; Mild aroma (76–90 points): Delicate rice fragrance; Faint aroma (61–75 points): Barely perceptible aroma; Neutral (36–60 points): No distinct fragrance but free from off-flavors; Off-flavored (0–35 points): Presence of undesirable odors. This study received ethical clearance from Henan University of Technology (Approval No. HAUTETHI–2023–07030154; Date: 3 July 2023).

### 2.5. Solvent Extraction of Volatile Compounds

A binary solvent system consisting of dichloromethane and anhydrous ether (1:1, *v*/*v*) was employed to extract volatile components from cooked rice [2,16]. After allowing the cooked rice (10 g) to rest for 10 min, it was rapidly frozen in liquid nitrogen and subsequently ground into a fine powder. A portion of the resulting rice flour (4 g) was mixed with 20 g of anhydrous sodium sulfate (Na_2_SO_4_) and stored overnight at −80 °C in an ultra-low temperature freezer. Following the freezing step, 4 g of this pretreated mixture was transferred to a conical flask, to which 15 mL of a 1:1 (*v*/*v*) solution of anhydrous ether and dichloromethane was added, along with 10 μL of internal standard (2-methyl-3-heptanone, 8.16 μg/mL).

To ensure efficient extraction, the sealed flask was shaken for 2 h at approximately 25 °C. The mixture was subjected to centrifugation at 3600× *g* for 5 min, and the resulting organic phase was collected and evaporated under nitrogen to a final volume of 1 mL.

### 2.6. Determination of Volatile Profiles Using GC–MS

A 1 μL portion of each sample was analyzed using a GC–MS system (Agilent 7890A/5975C, Agilent Technologies, Clara, CA, USA) with an HP–5MS column (30 m × 0.25 mm × 0.25 μm) for the separation and identification of volatiles. The injector operated in splitless mode and was maintained at 280 °C. High-purity helium (99.999%) served as the carrier gas at a fixed flow rate of 1.2 mL/min. The column temperature was programmed in several stages: initial hold at 40 °C for 1 min, ramped to 100 °C at 3 °C/min and held for 2 min; then increased to 160 °C at 3 °C/min and held for another 2 min; followed by a rise to 200 °C at the same rate and held for 2 min; finally, it was raised to 250 °C at 5 °C/min and held for 3 min. Electron impact ionization (EI) at 70 eV was applied for mass spectrometric detection. The GC–MS system was operated with the transfer line, quadrupole, and ion source held at 280 °C, 150 °C, and 230 °C, respectively. Mass spectra were recorded across a mass-to-charge (*m*/*z*) range of 35–450. The method was validated in terms of linearity, limits of detection and quantification (LOD/LOQ), recovery, and precision, and all parameters met the predefined acceptance criteria.

A homologous series of n-alkanes (C_5_–C_30_) was analyzed under identical chromatographic conditions to compute Kovats retention indices (RI). Tentative identification of volatiles was performed via comparison with the NIST 17 mass spectral database, accepting compounds with a matching score above 80% (Table S1). Further confirmation involved comparison of the measured RI with published literature values or data from authentic standards, when accessible. Relative contents of volatile compounds were determined semi-quantitatively via an internal standard (IS)-calibrated approach, utilizing the ratio between individual peak areas and the IS peak area [17].

### 2.7. GC–O Analysis

GC–MS analysis incorporated an olfactory detection port (ODP 3, GERSTEL, Mülheim an der Ruhr, Germany) to pinpoint odor-active constituents. Volatile compounds were collected using the extraction protocols detailed in Section 2.5 and Section 2.6. The effluent from the capillary column was divided equally between the mass spectrometer and the olfactory port (split ratio 1:1). Olfactory assessment was carried out by three trained assessors with prior experience in cereal aroma evaluation, who were selected and trained in line with ISO 8586:2023 and familiarized with artificial odorant standards. An odorant was considered detected if at least two evaluators simultaneously perceived it at the sniffing interface and gave comparable descriptions of its sensory attributes. The time at which each compound was perceived was recorded accordingly. This study received ethical clearance from Henan University of Technology (Approval No. HAUTETHI–2023–07030154; Date: 3 July 2023).

### 2.8. Calculation of OAV

OAV is a semi-quantitative index used to evaluate the contribution of an individual volatile compound to the overall aroma of a sample [18]. It is calculated as the ratio of the content of a specific volatile compound in the sample to its odor threshold in the same medium:

$${\mathrm{OAV}}_{i}\;=\;\frac{C_{i}}{{\mathrm{OT}}_{i}}$$

where *C_i_* is the concentration of compound *i*; *OT_i_* is the odor threshold of compound *i* in the same medium.

Given that cooked rice typically contains more than 50% water, the OAV for each volatile compound was obtained by calculating the ratio between its content and the corresponding odor threshold in aqueous media. This approach helps to estimate how strongly each compound contributes to the overall perceived aroma [19,20,21]. The threshold values were retrieved from relevant studies reported in the literature [3]. Odor thresholds reported in the literature may vary depending on the data source, experimental conditions, and matrix effects.

### 2.9. Statistical Analysis

To ensure data reliability, all tests were performed in triplicate. Statistical analyses were performed using SPSS (version 26) and GraphPad Prism (version 8.3). A one-way ANOVA was used to determine whether significant differences existed among sample means, with a 95% confidence interval. *K*-means clustering (k = 9) was performed on z-score–standardized contents of the volatile compounds [22,23]. The algorithm partitions variables by minimizing the within-cluster sum of squares (i.e., the sum of squared Euclidean distances to cluster centers) [24]. *K*-means clustering was implemented in R. In addition, data dimensionality and sample classification were explored through principal component analysis (PCA), hierarchical cluster analysis (HCA), and orthogonal partial least squares discriminant analysis (OPLS-DA) using the R package of MetaboAnalystR 4.0 [2,17]. HCA calculations employed Euclidean distance and Ward’s method for clustering [25]. OPLS-DA was used to maximize class separation by extracting a predictive component (T score [1]) correlated with Y and orthogonal component(s) (Orthogonal T score [1]) uncorrelated with Y [26]. Variable importance in projection (VIP) from OPLS-DA provided a useful criterion for identifying variables with high discriminatory potential [2]. VIP scores were calculated as a weighted sum of squares of the PLS loadings, incorporating the amount of Y-variance explained by each latent component [27]. Variables with VIP > 1 were considered important [25]. To identify discriminating volatiles between cultivars, pairwise comparisons were evaluated using a volcano plot approach that integrated two-sample *t*-tests with Benjamini–Hochberg false discovery rate (FDR) correction, fold-change (FC) analysis, and VIP scores from the OPLS-DA model. Specifically, *p*-values from the two-sample *t*-tests were adjusted using the Benjamini–Hochberg procedure to control the FDR, and FDR-adjusted *q* < 0.05 was considered statistically significant [28]. FC was calculated as the ratio of mean concentrations between two groups and log_2_(FC) was used for visualization in volcano plots [2]. Compounds meeting VIP > 1.0, FC > 1.2 or FC < 0.8, and FDR-adjusted *q* < 0.05 were defined as discriminating volatiles.

## 3. Results and Discussion

### 3.1. Odor Sensory Evaluation

**Figure 1:**
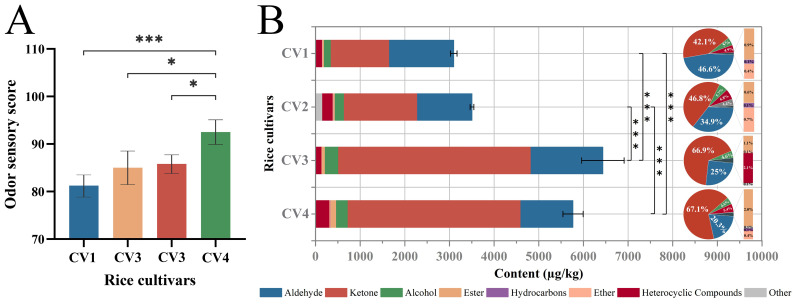
(**A**) Sensory evaluation results of odor in the four cultivars of cooked medium-milled fragrant Simiao rice (*: *p* < 0.05; ***: *p* < 0.001). (**B**) Total content of volatile compounds in the four cultivars of cooked medium-milled fragrant Simiao rice (***: *p* < 0.001), and the qualitative analysis of different volatile compound classes in the four cultivars of cooked medium-milled fragrant Simiao rice, expressed in the % pie chart.

Figure 1A shows the odor sensory evaluation outcomes for cooked medium-milled fragrant Simiao rice derived from four different cultivars. CV1 obtained the lowest aroma score, while CV2 and CV3 showed similarly low scores. No significant differences were observed in the aroma scores among CV1, CV2, and CV3 (*p* > 0.05), all of which exhibited a light cooked rice aroma. CV4, characterized by a strong cooked rice aroma, exhibited the highest aroma score, significantly higher than the other three samples (*p* < 0.05). Accordingly, Sample CV4 demonstrated the highest level of aroma acceptance among all tested samples.

### 3.2. Volatile Compound in the Cooked Rice

#### 3.2.1. Identification of Volatile Compounds

The overall aroma of rice was largely dictated by the composition and content of its volatile compounds. Therefore, the volatile compounds in the four cultivars of cooked medium-milled fragrant Simiao rice were identified by GC–MS. As shown in Table 1, a total of 45 volatiles were detected and categorized into 8 types: 15 aldehydes, 8 ketones, 5 alcohols, 6 esters, 2 hydrocarbons, 2 ethers, 5 heterocycles, and two compounds classified as ‘others’. The total content of volatile compounds was higher in CV3 and CV4 than in CV1 and CV2 (*p* < 0.001) (Figure 1B). The higher presence of acetoin in CV3 and CV4 possibly contributes to this observation (Table 1). Moreover, there was no significant difference in total volatile compound content between CV3 and CV4 (*p* > 0.05; Figure 1B). The majority of the volatile compounds in CV1 were aldehydes, followed by ketones and alcohols, accounting for 46.6%, 42.1%, and 5.0% of the total volatile compounds, respectively. In CV2, CV3, and CV4, ketones were the majority of the volatile compounds, followed by aldehydes and then alcohols, comprising 46.8–67.1%, 20.3–34.9%, and 4.5–5.7% of the total volatile compounds, respectively. Table 1 shows the content (µg/kg) and odor threshold (µg/kg) in water of volatile compounds identified in the four cultivars of cooked rice.

#### 3.2.2. Comparison of Volatile Compounds

**Figure 2:**
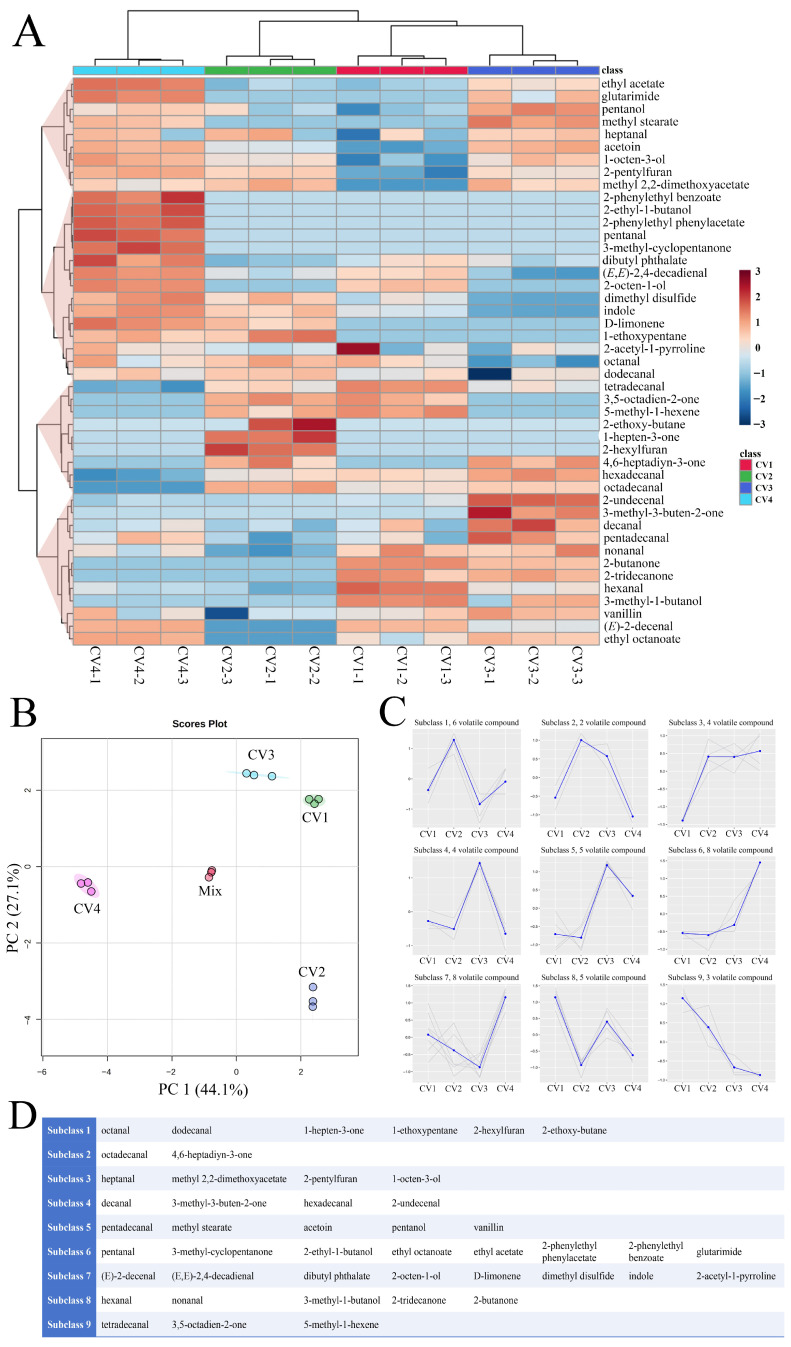
(**A**) Quantitative analysis of volatile compounds exhibited in heatmaps. The colors of the heatmap indicated the relative content of each volatile compound, from low (blue) to high (red). (**B**) PCA score plot of volatile compounds identified in the four cultivars of cooked medium-milled fragrant Simiao rice. (**C**) *K*-means clustering groups of the expression profile of the volatile compounds in the four cultivars of cooked medium-milled fragrant Simiao rice. The Y axis represented the standardized content of each volatile compound, and the X axis represented the different rice cultivars. In panel (**C**), grey lines represent individual compounds within each subclass, and the blue line indicates the subclass mean trend. (**D**) Summary of *K*-means-derived subclasses for volatile compounds.

Multivariate statistical analyses were employed to assess differences in volatile profiles across the four rice cultivars. The 3 biological replicates for each sample clustered closely together in both HCA and PCA analyses (Figure 2A,B), suggesting minimal variation among core samples within their respective groups, thereby demonstrating the reproducibility and reliability of the experiment [38]. HCA revealed that CV4 clearly separated from the other three cultivars, suggesting a unique volatile profile. Moreover, CV1 and CV3 clustered together due to their high similarity. This clustering pattern was further corroborated by the PCA score plot (Figure 2B). Principal component 1 (PC 1) and principal component 2 (PC 2) explained 44.1% and 27.1% of the total variance, respectively, resulting in a combined contribution of 71.2% (Figure 2B). The quality control samples, referred to as Mix in Figure 2B, clustered closely together in the PCA score plot. This suggests that they possess similar volatile profiles and confirms the stability and repeatability of the analysis [39]. The analysis partitioned the samples into three distinct groups, each characterized by its own specific volatile profile. Group 1 consisted exclusively of CV4, and Group 2 consists solely of CV2. Group 3 consists of CV1 and CV3, indicating that CV1 and CV3 had similar volatile profiles. The HCA and PCA results indicated that CV4 exhibited distinct volatile profiles compared to CV1, CV2, and CV3. To further elucidate the specific chemical distribution patterns driving these cultivar differences, *K*-means clustering was applied to the standardized volatile data. This analysis successfully classified the 45 volatile compounds into 9 distinct subclasses (Figure 2C,D). The volatile compounds belonging to the same subclass exhibited similar distribution trends in all samples.

#### 3.2.3. Identification of Volatile Markers

**Figure 3:**
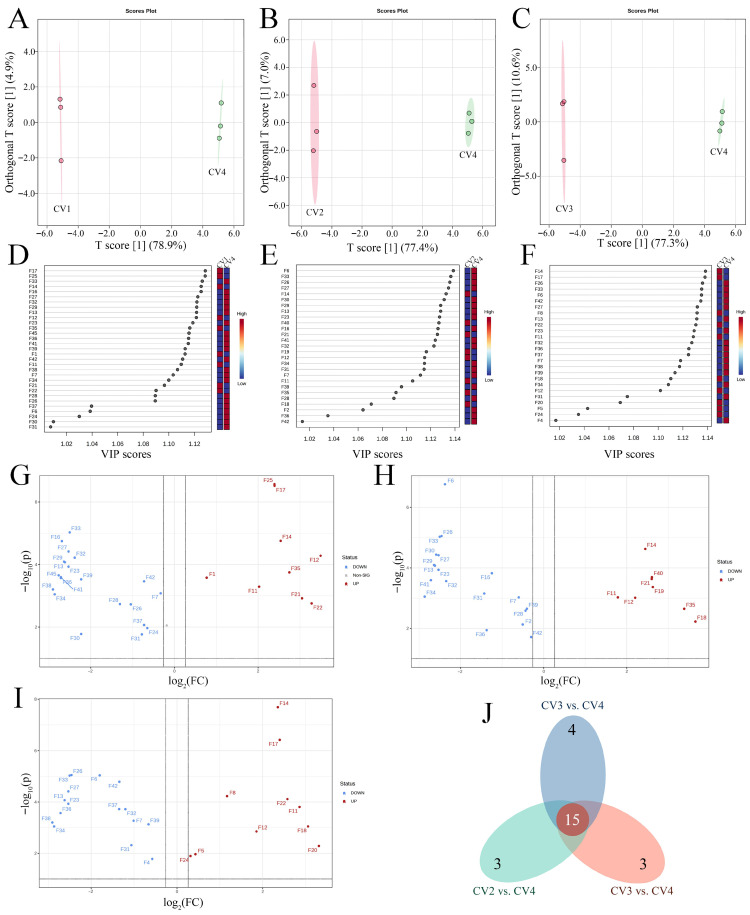
(**A**)–(**C**) OPLS-DA model plot between CV1 and CV4 (**A**), CV2 and CV4 (**B**), and CV3 and CV4 (**C**). Points are colored by group, and the shaded ellipses indicate the 95% confidence region. T score [1] and Orthogonal T score [1] denote the predictive and orthogonal components of the OPLS-DA model, respectively. (**D**–**F**) The volatile compounds were ranked according to their variable importance for the projection (VIP) scores obtained from OPLS-DA. Only volatile compounds with a VIP score greater than 1.0 were included. (**G**–**I**) Volcano plots of the volatile compounds between CV1 and CV4 (**G**), CV2 and CV4 (**H**), and CV3 and CV4 (**I**). Blue spots show downregulated differentially expressed volatile compounds; red spots illustrate upregulated differentially expressed volatile compounds; gray spots represent detected volatile compounds with insignificant differences. The vertical and horizontal dashed lines indicate the thresholds for differential expression (|log2(FC)| cutoff) and statistical significance (*p*-value cutoff), respectively. (**J**) Venn diagram showing the overlapping and specific discriminating volatiles. Detailed compound code specifications are provided in Table 1.

Based on its superior odor sensory scores and distinct volatile profile, OPLS-DA models were independently constructed to compare CV4 with each of the other rice cultivars (CV1, CV2, and CV3). This approach was employed to identify the discriminating volatiles underlying for the observed differences. In the OPLS-DA score plots, T score [1] (predictive component) represented X-variation correlated with class membership (Y), whereas Orthogonal T score [1] captured systematic X-variation orthogonal to Y and was commonly interpreted as within-class (structured) variation [26]. In the OPLS-DA models, CV4 was clearly distinguished from CV1, CV2, and CV3, indicating significant differences in their volatile compound profiles (Figure 3A–C). The cross-validation results (1000 permutations) demonstrated the robustness of the OPLS-DA models (Figure S1). To evaluate the content of specific volatile compounds in differentiating CV4 from CV1, CV2, and CV3, the VIP scores derived from the OPLS-DA model were employed. A threshold of VIP > 1.0 was used to screen volatile compounds that significantly contributed to the classification of the samples (Figure 3D–F). These discriminative volatiles, identified via OPLS-DA, were further evaluated using statistical tests, including two-sample *t*-tests corrected by the Benjamini-Hochberg procedure for false discovery rate (FDR) and fold-change (FC) analysis (Figure 3G–I). Ultimately, compounds meeting the criteria of VIP > 1.0, FC > 1.2 or <0.8, and FDR-adjusted *q* < 0.05 were classified as discriminating volatiles. A total of 39 discriminating volatile compounds were identified across 8 categories, including 11 aldehydes, 8 ketones, 5 alcohols, 6 esters, 2 hydrocarbons, 2 ethers, 4 heterocyclic compounds, and 1 compound classified as “other” (Figure 3). Among them, only 6 compounds (heptanal, dodecanal, pentadecanal, vanillin, 2-acetyl-1-pyrroline, and 2-ethoxy-butane) were not identified as discriminating volatiles. These results strongly indicate that CV4 differs significantly from the other three samples in both the types and contents of volatile compounds. Specifically, 30, 26, and 27 discriminating volatiles were identified in the comparison groups CV1 vs. CV4, CV2 vs. CV4, and CV3 vs. CV4, respectively. The discriminating volatiles mainly originated from lipid oxidation, particularly the thermo-oxidation of unsaturated fatty acids (e.g., pentanal, hexanal, and (*E*,*E*)-2,4-decadienal), amino acid degradation and Strecker reactions (e.g., 2-ethyl-1-butanol, indole, and dimethyl disulfide), as well as esterification and carbonyl-related reactions and minor residues of plant secondary metabolites (e.g., ethyl acetate, 2-phenylethyl benzoate, and d-limonene) [10]. Therefore, the differences in volatile profiles between CV4 and the other cultivars might have been associated with variations in precursor pools and reaction sensitivities. Moreover, 15 discriminating volatiles were consistently present in all 3 comparison groups simultaneously, including 5 aldehydes ((*E*,*E*)-2,4-decadienal, hexadecanal, tetradecanal, pentanal, octadecanal), 1 ketone (3-methyl-cyclopentanone), 2 alcohols (2-octen-1-ol and 2-ethyl-1-butanol), 4 esters (dibutyl phthalate, ethyl acetate, 2-phenylethyl phenylacetate, and 2-phenylethyl benzoate), 1 hydrocarbon (d-limonene), 2 heterocyclic compounds (2-pentylfuran and indole) (Figure 3J). Among these, 12 discriminating volatiles ((*E*,*E*)-2,4-decadienal, pentanal, 3-methyl-cyclopentanone, 2-octen-1-ol, 2-ethyl-1-butanol, dibutyl phthalate, ethyl acetate, 2-phenylethyl phenylacetate, 2-phenylethyl benzoate, d-limonene, 2-pentylfuran, and indole) showed obviously higher content in CV4, suggesting their potential role as characteristic volatile markers for CV4.

### 3.3. The Odor of Cooked Rice

The overall contribution of a volatile compound to food aroma was influenced by both its content and its odor threshold [20,40]. Therefore, after the discriminating volatiles between CV4 and the other samples were identified, the potential reasons behind the higher aroma scores of CV4 were further investigated by incorporating the odor threshold and GC–O results.

#### 3.3.1. OAVs of Volatile Compounds

**Figure 4:**
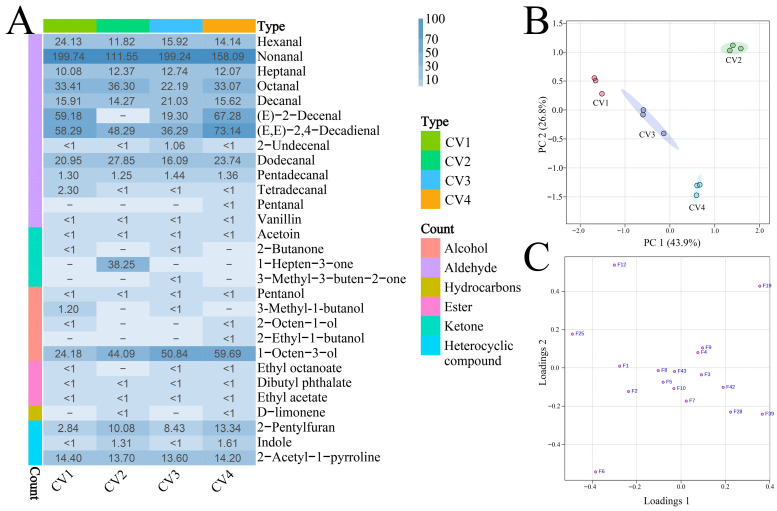
(**A**) The OAVs of 29 volatile compounds in the four cultivars of cooked medium-milled fragrant Simiao rice. (**B**) The PCA score plot and (**C**) the PCA loading plot were constructed using the 17 key odor-active compounds. Detailed compound code specifications are provided in Table 1.

The OAVs of 29 volatile compounds with documented odor thresholds were calculated, allowing for a systematic assessment of their contributions to the overall aroma profiles of the different rice cultivars (Table 1 and Figure 4A). Aldehydes were identified as the predominant contributors among the high-OAV volatiles, underscoring their importance in the aroma profile of the cooked rice samples (Figure S2). Among the identified volatiles, nonanal emerged as the dominant contributor to the aroma across all four rice cultivars (Figure S2). Nonanal, characterized by citrusy, green, and fatty odor, played a pivotal role in the aroma perception of the rice samples [30,41]. It was widely believed that nonanal contributes to the pleasant aroma of rice [6]. All samples contained appreciable content of ketones, mainly acetoin and 2-butanone, both of which have high odor thresholds (Table 1). Nevertheless, their content in this study was below their respective odor thresholds, suggesting limited contributions to overall odor.

#### 3.3.2. Identification of Key Odor-Active Compounds

The volatile compounds with an OAV greater than 1.0 and detectable by GC–O were considered to have key odor activity and were important for the overall odor profile of the sample [20]. Of the 29 volatile compounds in Section 3.3.1, 17 compounds demonstrated OAV greater than 1.0 and were also detected via GC–O. Hence, these compounds were classified as key odor-active compounds [22,42]. They encompassed a diverse set of chemical classes, including a majority of aldehydes, predominantly ranging from C6 to C15 linear or unsaturated chains (e.g., hexanal, (*E*,*E*)-2,4-decadienal, dodecanal), along with a ketone (1-hepten-3-one), 3 heterocyclic compounds (2-acetyl-1-pyrroline, indole, and 2-pentylfuran), and 2 alcohols (3-methyl-1-butanol and 1-octen-3-ol) (Figure 4A). Moreover, a subset of compounds, including (*E*,*E*)-2,4-decadienal, hexanal, heptanal, decanal, dodecanal, pentadecanal, nonanal, 1-hepten-3-one, octanal, 2-pentylfuran, and 2-acetyl-1-pyrroline, exhibited OAV > 1.0 across all examined samples. Although the OAV and relative proportions of them in the four samples were different, they accounted for 81.3% to 91.2% of the total OAV of all volatile compounds in these samples (Figure S2). Therefore, these compounds played a decisive role in shaping the aroma profiles of the four cooked medium-milled fragrant Simiao rice.

#### 3.3.3. Comparison of Key Odor-Active Compounds

Although the content of 2-acetyl-1-pyrroline was commonly used as an aroma indicator for selecting aromatic lines, variations in 2-acetyl-1-pyrroline content alone failed to adequately explain the differences in odor among different cultivars of fragrant rice. This could be attributed to the fact that the aroma quality of rice is governed by the synergistic interaction of multiple key volatile compounds rather than by any single compound alone [10]. Therefore, a comprehensive analysis of key odor-active compounds was essential to differentiate variations between offspring in rice breeding programs. PCA based on the OAVs of the 17 key odor-active compounds across the four rice samples was illustrated in Figure 4B,C. The principal components 1 (PC 1) and 2 (PC 2) explained 43.9% and 26.8% of the variance, respectively, accounting for 70.7% in total. The PCA score plot clearly illustrated the distinct distribution of the four samples (CV1, CV2, CV3, and CV4) in separate areas, implying significant differences in the type and/or content of their key odor-active compounds (Figure 4B). This variation in key odor-active compounds would result in notable odor differences among the four samples, corroborating the odor sensory evaluation results described in Section 3.1. The PCA loading plots illustrated the key odor-active compounds that drove the separation of these four samples (Figure 4C).

Hexanal, nonanal, tetradecanal, and 3-methyl-1-butanol were associated with CV1 in the loading plot (Figure 4C). Hexanal, known for its fatty, oily, grassy, and green odor notes, was commonly associated with undesirable aromas caused by lipid oxidation in rice [6]. A high content of hexanal had the potential to negatively affect consumer acceptance. Nonanal was identified as a key compound contributing to the overall aroma and sensory characteristics of rice [6,41], with a pleasant scent that was described as citrusy, green, and fatty [3]. Tetradecanal imparted waxy and fatty aroma attributes, whereas 3-methyl-1-butanol was characterized by a fruity odor. Meanwhile, tetradecanal and 3-methyl-1-butanol were only present in CV1 with OAV > 1.0 (Figure 4A). Therefore, CV1 might exhibit more pronounced citrusy, fatty, green, and fruity odor characteristics. The relatively high content of hexanal appeared to have a negative impact on its overall odor profile.

As shown in Figure 4C, octanal, dodecanal, and 1-hepten-3-one were associated with CV2. Among them, octanal was particularly noteworthy, as it had been widely recognized as a key marker of early-stage rice oxidation and was closely linked to the formation of off-flavors during storage [3]. Sensory descriptors for octanal commonly included grassy and oily odor. 1-hepten-3-one and dodecanal exhibited distinct odor characteristics, with the former contributing metallic notes and the latter imparting a minty odor [43], and they were only present in CV2 with OAV > 1.0 (Figure 4A). The co-occurrence of these odor-active compounds suggested that CV2 possessed more prominent grassy, oily, metal, and minty odor characteristics than other cultivars. Meanwhile, the elevated content of octanal might have been a key factor contributing to its relatively lower aroma score.

Hexanal, nonanal, decanal, 2-undecenal, and pentadecanal were associated with CV3 (Figure 4C). Decanal was widely regarded as an important contributor to the aromatic characteristics of rice [44]. Its odor was described as pleasant, with green, citrus, sweet, floral, and soapy notes [32,33]. 2-undecenal was associated with sweet and fatty odor characteristics, and pentadecanal exhibited fresh and citrus odor characteristics. Although the higher content of decanal and nonanal in CV3 contributed to a pleasant aroma, the relatively high level of hexanal present in the sample might limit its acceptance in the consumer market.

(*E*,*E*)-2,4-decadienal, 1-octen-3-ol, indole, 2-pentylfuran were associated with CV4 (Figure 4C). (*E*,*E*)-2,4-decadienal was characterized by its fatty, waxy, nutty, and melon odor, and it contributed significantly to the aroma of rice [45,46,47]. 1-octen-3-ol exhibited a distinct raw mushroom-like odor and was previously reported as a contributing odor compound in various fragrant rice cultivars [6,41]. Conversely, Gao et al. suggested that this compound was responsible for the undesirable odor associated with rice bran [30]. The content of 2-pentylfuran strongly affects its odor profile [30]. At lower content, 2-pentylfuran emitted a noticeable nutty aroma, whereas at higher content, it produced an unpleasant odor reminiscent of soy [3,48]. These findings suggested that the perceived odor of 2-pentylfuran was highly concentration-dependent. As a result, some studies emphasized 2-pentylfuran as a crucial component in generating desirable fragrances [49], whereas others highlighted its role as the primary factor responsible for the production of unpleasant odors [32,50]. Therefore, the roles of 1-octen-3-ol and 2-pentylfuran in the formation of the odor in cooked rice still require further investigation. Indole, a common volatile compound detected in cooked rice, contributed to its aroma by imparting floral, fruity, and herbaceous notes, even at low content [2,17]. The higher content of (*E*,*E*)-2,4-decadienal and indole in sample CV4 contributed to its enhanced odor quality, thereby increasing its aroma acceptance. Moreover, the concentrations of hexanal and octanal, compounds known to affect rice aroma negatively, were significantly lower in CV4, effectively reducing off-flavor interference and helping to highlight its characteristic aroma.

#### 3.3.4. Identification of Odor Characteristics of Xiangzhuxiang

Of the 39 discriminating volatiles identified in Section 3.2.3, 14 compounds were classified as key odor-active compounds, including 9 aldehydes ((*E*,E)-2,4-decadienal, pentadecanal, nonanal, (*E*)-2-decenal, octanal, tetradecanal, 2-undecenal, hexanal, and decanal), 1 ketone (1-hepten-3-one), 2 heterocyclic compounds (2-pentylfuran and indole), and 2 alcohols (1-octen-3-ol and 3-methyl-1-butanol). Unlike the other discriminating volatiles, these 14 compounds were likely to be detectable by humans, as indicated by their OAVs > 1.0 and confirmation through GC-O analysis. Although the OAVs and relative proportions varied among the four samples, these compounds collectively accounted for 90.8%, 85.4%, 90.0%, and 89.3% of the total OAV of all volatiles detected in samples CV1, CV2, CV3, and CV4, respectively. Therefore, the presence of these compounds played a crucial role in accounting for the distinct odor profiles observed between CV4 and the other three cultivars. These discriminating volatiles were defined as discriminating key odor-active compounds.

**Figure 5:**
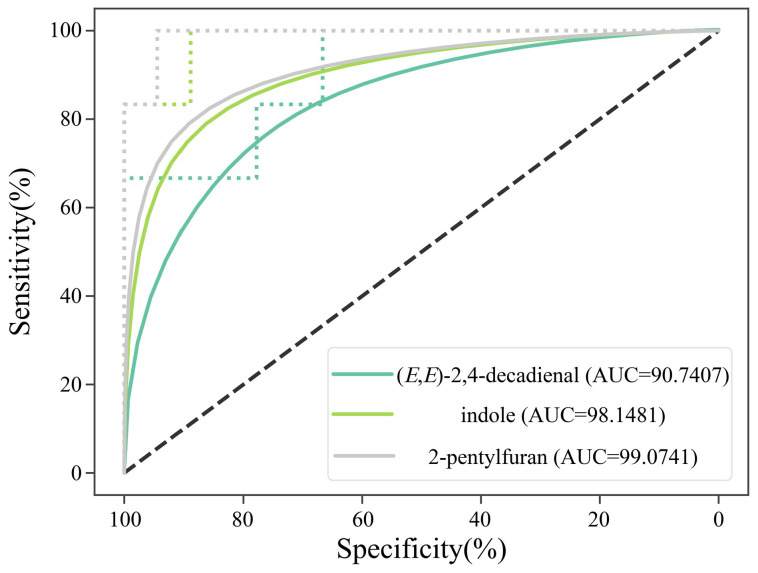
Receiver operating characteristic (ROC) curves of (*E*,*E*)-2,4-decadienal, 2-pentylfuran and indole for discriminating the CV4 from the other three fragrant Simiao rice cultivars. Area under the curve (AUC) values are shown in the legend, and the dashed diagonal line represents random classification (AUC = 0.5).

In the pairwise comparison of CV1 vs. CV4, CV2 vs. CV4, and CV3 vs. CV4, totals of 7, 8, and 8 discriminating key odor-active compounds were identified, respectively. Specifically, in the comparison of CV1 vs. CV4, 4 compounds were found at higher content (FC > 1.2) in CV4 (2-pentylfuran, 1-octen-3-ol, indole, and (*E*,*E*)-2,4-decadienal), while 3 compounds exhibited higher content (FC > 1.2) in CV1 (hexanal, tetradecanal, and 3-methyl-1-butanol). In the comparison of CV2 vs. CV4, 6 compounds ((*E*,*E*)-2,4-decadienal, nonanal, 1-octen-3-ol, (*E*)-2-decenal, indole, and 2-pentylfuran) had higher content (FC > 1.2) in CV4, whereas 2 (tetradecanal and 1-hepten-3-one) were more abundant (FC > 1.2) in CV2. Similarly, the comparison of CV3 vs. CV4 revealed 5 compounds ((*E*,*E*)-2,4-decadienal, octanal, 2-pentylfuran, (*E*)-2-decenal, and indole) at higher content (FC > 1.2) in CV4, and 3 compounds (decanal, 2-undecenal, and tetradecanal) at higher content (FC > 1.2) in CV3. There were 4 discriminating key odor-active compounds present in all 3 comparison groups, namely (*E*,*E*)-2,4-decadienal, tetradecanal, 2-pentylfuran, and indole. Among these, (*E*,*E*)-2,4-decadienal, 2-pentylfuran, and indole exhibited higher content (FC > 1.2) in CV4 compared to the other three samples (CV1, CV2, and CV3), whereas tetradecanal displayed the opposite trend. To further validate the ability of (*E*,*E*)-2,4-decadienal, 2-pentylfuran, and indole to discriminate CV4 from the other three cultivars (CV1 to CV3), a receiver operating characteristic (ROC) analysis was performed (Figure 5). The results demonstrated that all three biomarkers exhibited excellent diagnostic performance with area under the curve (AUC) values exceeding 0.90. Specifically, 2-pentylfuran showed the highest predictive accuracy (AUC = 0.991), followed by indole (AUC = 0.981) and (*E*,*E*)-2,4-decadienal (AUC = 0.907). Collectively, the statistical validations, combined with their high OAVs, confirm that (*E*,*E*)-2,4-decadienal, 2-pentylfuran, and indole significantly contributed to the formation of the distinct odor characteristics associated with CV4. However, given the pronounced concentration-dependent behavior of 2-pentylfuran and the fact that the critical concentration at which its odor shifts from pleasant to unpleasant has not yet been clearly defined, the specific contribution of 2-pentylfuran to the aroma of CV4 warrants further in-depth investigation. Nonetheless, it could be clearly indicated that the higher aroma score of CV4 was directly associated with its elevated content of (*E*,*E*)-2,4-decadienal and indole. These compounds contribute significantly to the nutty, fatty, and sweet aroma characteristics of CV4.

## 4. Conclusions

This study systematically elucidated the volatile and key odor-active compound profiles of cooked medium-milled rice from four major fragrant Simiao rice cultivars grown in Southern China, identifying the critical components driving their sensory differences. A total of 45 volatile compounds and 17 key odor-active compounds were detected across the four samples, 11 of which were shared by all cultivars. Consequently, despite varietal differences, the four samples exhibited similar key odor-active compound profiles, with nonanal constituting the core component (contributing 29.9–47.2% to the total OAV). Odor sensory evaluation showed that the cultivar “Xiangzhuxiang” (CV4) obtained significantly higher odor sensory scores than the other three cultivars. However, differences in nonanal content alone could not account for the significant differences in odor sensory scores. Integrated analysis of OAV, GC–O, and multivariate statistics indicated that the superiority of Xiangzhuxiang (CV4) mainly resulted from the enrichment of pleasant odorants and the attenuation of undesirable ones. Specifically, (*E*,*E*)-2,4-decadienal and indole were markedly enriched in Xiangzhuxiang (VIP > 1.0, FC > 1.2, FDR-adjusted *q* < 0.05), enhancing the overall aroma quality with roasted nutty, fatty, floral, and sweet notes. In contrast, hexanal and octanal were maintained at moderate levels, thereby mitigating the rancid notes associated with lipid oxidation. Therefore, improving the aroma quality of medium-milled fragrant Simiao rice should focus on regulating lipid-oxidation-derived volatile compounds.

## Figures and Tables

**Table 1:** The content (µg/kg) and odor threshold (µg/kg) in water of volatile compounds identified in the four cultivars of cooked rice.

NO.	Volatile Compound ^a^	Odor description		Odor Threshold (µg/kg) ^d^	Relative Content (μg/kg) ^e^				RI		Identification Method ^h^
		Literature ^b^	Experiment ^c^		CV1	CV2	CV3	CV4	Experiment ^f^	Literature ^g^	
Aldehyde (15)											
F1	hexanal	fatty, oily, grass, green	fatty, green	4.5	109	53.2	71.6	63.6	791	800	MS, RI, STD, O
F2	nonanal	green, citrus	green, citrus	1	200	112	199	158	1100	1102	MS, RI, STD, O
F3	heptanal	fresh, fruity-like, floral	sweet, fresh, fruity-like	3	30.2	37.1	38.2	36.2	895	899	MS, RI, STD, O
F4	octanal	fatty, grassy, lemon, green, fruity	fatty, grassy	0.7	23.4	25.4	15.5	23.2	982	981	MS, RI, STD, O
F5	decanal	green, citrus, sweet, floral, soapy	citrus, floral, soapy	3.02	48.1	43.1	63.5	47.2	1204	1203	MS, RI, STD, O
F6	(*E*)-2-decenal	green, waxy, fatty	waxy, fatty	0.4	23.7	n.d.	7.72	26.9	1262	1265	MS, RI, STD, O
F7	(*E*,*E*)-2,4-decadienal	fatty, waxy, nutty, melon, citrus	nutty, fatty	0.07	4.08	3.38	2.54	5.12	1319	1320	MS, RI, STD, O
F8	2-undecenal	sweet, fatty	sweet, fatty	340	180	166	360	160	1365	1359	MS, RI, O
F9	dodecanal	minty, soapy	minty	1.07	22.4	29.8	17.2	25.4	1409	1412	MS, RI, STD, O
F10	pentadecanal	fresh	fresh, citrus	430	559	536	619	587	1719	1715	MS, RI, O
F11	hexadecanal	-	-	-	53.6	45.5	95.6	13.2	1820	1822	MS, RI, STD
F12	tetradecanal	waxy, floral	waxy, fat	60	138	56.4	44.3	12.3	1830	1822	MS, RI, STD, O
F13	pentanal	-	-	12	n.d.	n.d.	n.d.	6.84	701	698	MS, RI
F14	octadecanal	-	-	91	47.4	113	69.4	n.d.	2027	2024	MS, RI
F15	vanillin	-	-	58	3.98	2.43	6.99	4.06	1395	1394	MS, RI, STD
Ketone (8)											
F16	acetoin	-	-	8000	598	1630	3900	3870	710	720	MS, RI, STD
F17	2-butanone	-	-	3000	696	n.d.	399	n.d.	600	602	MS, RI
F18	4,6-heptadiyn-3-one	-	-	-	n.d.	4.33	4.52	n.d.	862	-	MS
F19	1-hepten-3-one	geranium-like	geranium-like, sweet	0.04	n.d.	1.53	n.d.	n.d.	856	856	MS, RI, O
F20	3-methyl-3-buten-2-one	-	-	1000	n.d.	n.d.	2.75	n.d.	660	653	MS, RI
F21	3,5-octadien-2-one	-	-	-	6.13	6.87	n.d.	n.d.	1099	1093	MS, RI
F22	2-tridecanone	-	-	-	3.63	n.d.	3.09	n.d.	1489	1494	MS, RI, STD
F23	3-methyl-cyclopentanone	-	-	-	n.d.	n.d.	n.d.	1.21	850	847.5	MS, RI
Alcohol (5)											
F24	pentanol	-	-	4000	126	157	245	197	771	768	MS, RI, STD
F25	3-methyl-1-butanol	fruity, malty	fruity	4.0	4.78	n.d.	1.23	n.d.	783	779	MS, RI, STD, O
F26	2-octen-1-ol	-	-	50	1.80	n.d.	n.d.	3.71	1041	1039.7	MS, RI
F27	2-ethyl-1-butanol	-	-	75.2	n.d.	n.d.	n.d.	1.42	830	830	MS, RI
F28	1-octen-3-ol	straw, earthy, raw mushroom	raw mushroom	1	24.2	44.1	50.8	59.7	970	976	MS, RI, STD, O
Ester (6)											
F29	methyl stearate	-	-	-	n.d.	n.d.	20.2	10.4	2133	2130	MS, RI, STD
F30	ethyl octanoate	-	-	650	3.15	n.d.	9.35	14.8	1195	1196	MS, RI, STD
F31	dibutyl phthalate	-	-	260	12.3	7.83	10.1	21.1	1964	1959.7	MS, RI, STD
F32	ethyl acetate	-	-	100	12.4	12.7	28.1	64.8	515	515	MS, RI, STD
F33	2-phenylethyl phenylacetate	-	-	-	n.d.	n.d.	n.d.	4.73	1926	1924.3	MS, RI
F34	2-phenylethyl benzoate	-	-	-	n.d.	n.d.	n.d.	1.57	1863	1858.9	MS, RI
Hydrocarbons (2)											
F35	5-methyl-1-hexene	-	-	-	3.65	1.96	n.d.	n.d.	653	652	MS, RI
F36	d-limonene	-	-	34	n.d.	2.60	n.d.	6.74	1042	1044	MS, RI, STD
Ether (2)											
F37	dimethyl disulfide	-	-	-	13.6	18.3	8.9	22.6	741	740	MS, RI
F38	1-ethoxypentane	-	-	-	n.d.	5.44	n.d.	3.00	793	788	MS, RI
Heterocyclic Compounds (5)											
F39	2-pentylfuran	green, nutty, bean, beany	bean	4.8	13.7	48.4	40.5	64.1	968	970	MS, RI, STD, O
F40	2-hexylfuran	-	-	-	n.d.	5.49	n.d.	n.d.	1100	1096	MS, RI
F41	glutarimide	-	-	-	n.d.	n.d.	2.30	18.6	1156	1153.9	MS, RI, STD
F42	indole	sweet, burnt, floral	sweet	140	136.4	183.1	88.9	225.8	1290	1293	MS, RI, STD, O
F43	2-acetyl-1-pyrroline	pandan, cooked rice, sweet, pleasant, popcorn	popcorn, cooked rice	0.1	1.44	1.37	1.36	1.42	930	922	MS, RI, STD, O
Other (2)											
F44	2-ethoxy-butane	-	-	-	n.d.	143	n.d.	n.d.	635	622	MS, RI
F45	methyl 2,2-dimethoxyacetate	-	-	-	n.d.	11.6	9.45	7.08	504	-	MS

^a^ Only identified compounds were shown. ^b^ Odor description reported in the literature [2,3,6,17,25,29,30,31,32,33,21]. ^c^ Odor description was obtained from GC–O analysis and was verified by the standard odorant and in accordance with the book from Burdock (2009) [34]. ^d^ Odor threshold (µg/kg) according to previous studies [3,29,35,36]. ^e^ Relative content was semi-quantified using 2-methyl-3-heptanone as the internal standard, based on the ratio of the peak area of each compound to that of the internal standard. Results were reported to three significant figures. ^f^ Experimental Kovat’s index calculated from volatile compounds on an HP–5MS capillary column with a homologous series of n-alkanes (C_5_–C_30_). ^g^ The published Kovats index according to the Cornell University Flavornet database (http://www.flavornet.org/flavornet.html (accessed on 23 November 2022)), NIST (https://www.nist.gov/ (accessed on 23 November 2022)), and published literature [12,16,29,30,31,37], in which the columns had similar polarity to this investigation. ^h^ Identification methods: MS, mass spectra; RI, retention index; STD, standard chemical; O, GC–O.

## Data Availability

The original contributions presented in this study are included in the article/Supplementary Materials. Further inquiries can be directed to the corresponding authors.

## Supplementary Materials

The following supporting information can be downloaded at: https://www.mdpi.com/article/10.3390/foods15020356/s1, Figure S1: The model cross-validation parameters (1000 times) of the comparison groups of CV1 vs. CV4 (A), CV2 vs. CV4 (B), and CV3 vs. CV4 (C) (CV1 vs. CV4: R^2^Y = 0.999 and Q^2^ = 0.999; CV2 vs. CV4: R^2^Y = 0.997 and Q^2^ = 0.995; CV3 vs. CV4: R^2^Y = 0.999 and Q^2^ = 0.999); Figure S2: The proportional contribution of OAVs of volatile compounds in the four cultivars of cooked medium-milled fragrant Simiao rice, expressed in the % doughnut chart; Table S1. Match factor and quantifier ion (*m*/*z*) of volatile compounds.
