# Supplementary material for: The Preferred Odor Characteristics of Cooked Medium-Milled Fragrant Simiao Rice
Source: Foods. 2026 Jan 19;15(2):356. doi: 10.3390/foods15020356 (PMC12840958; doi:10.3390/foods15020356)
Supplement: Supplementary file 1 [file foods-15-00356-s001.zip › foods-4042167-supplementary.pdf]

The preferred odor characteristics of cooked medium-milled fragrant Simiao rice

Supplementary Material

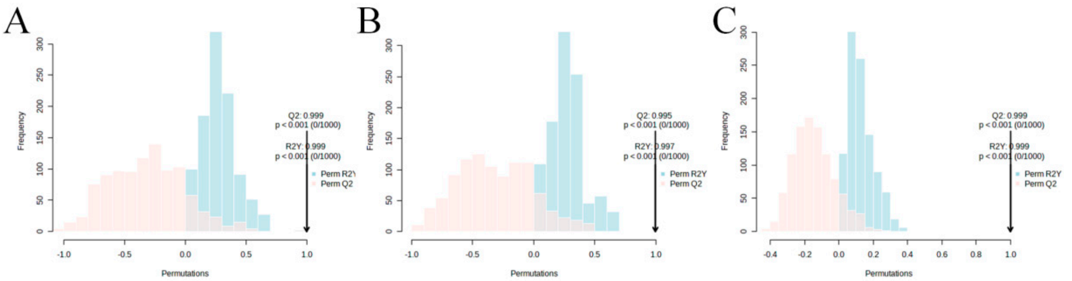

Figure S1 The model cross-validation parameters (1000 times) of the comparison groups of CV1 vs. CV4 (A), CV2 vs. CV4 (B), and CV3 vs. CV4 (C) (CV1 vs. CV4: R^2Y = 0.999 and Q^2 = 0.999; CV2 vs. CV4: R^2Y = 0.997 and Q^2 = 0.995; CV3 vs. CV4: R^2Y = 0.999 and Q^2 = 0.999).

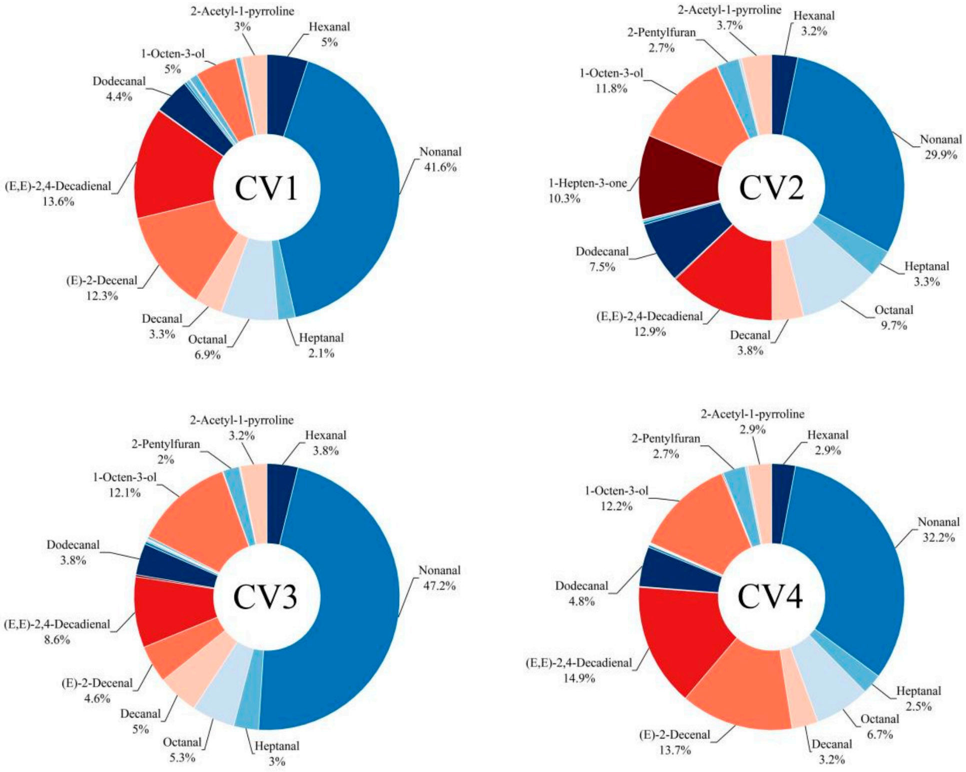

Figure S2 The proportional contribution of OAVs of volatile compounds in the four cultivars of cooked medium-milled fragrant Simiao rice, expressed in the % doughnut chart.

Table S1. Match factor and quantifier ion (m/z) of volatile compounds.

NO.	Volatile compound	Match factor	m/z	NO.	Volatile compound	Match factor	m/z
F1	hexanal	92.8	44	F24	pentanol	82.7	55
F2	nonanal	95.9	57	F25	3-methyl-1-butanol	84.6	55
F3	heptanal	86.4	44	F26	2-octen-1-ol	83.2	57
F4	octanal	91.6	57	F27	2-ethyl-1-butanol	83.2	57
F5	decanal	96.5	57	F28	1-octen-3-ol	83	57
F6	(E)-2-decenal	91.3	55	F29	methyl stearate	89.6	74
F7	(E,E)-2,4-decadienal	92.7	81	F30	ethyl octanoate	84.3	88
F8	2-undecenal	96.4	55	F31	dibutyl phthalate	88.4	149
F9	dodecanal	87.2	57	F32	ethyl acetate	82.7	43
F10	pentadecanal	86.9	57	F33	2-phenylethyl phenylacetate	85.4	91
F11	hexadecanal	91.6	57	F34	2-phenylethyl benzoate	97.5	91
F12	tetradecanal	85.5	57	F35	5-methyl-1-hexene	82.1	41
F13	pentanal	87.7	44	F36	D-limonene	88.5	68
F14	octadecanal	92.3	57	F37	dimethyl disulfide	82.5	94
F15	vanillin	89.4	151	F38	1-ethoxypentane	97.4	45
F16	acetoin	86.2	45	F39	2-pentylfuran	89.9	81
F17	2-butanone	97.5	43	F40	2-hexylfuran	81.3	81
F18	4,6-heptadiyn-3-one	82.1	79	F41	glutarimide	85.3	84
F19	1-hepten-3-one	94.8	55	F42	indole	88.1	117
F20	3-methyl-3-buten-2-one	82.2	43	F43	2-acetyl-1-pyrroline	91.8	111
F21	3,5-octadien-2-one	85.9	81	F44	2-ethoxy-butane	81.5	45
F22	2-tridecanone	83.5	58	F45	methyl 2,2-dimethoxyacetate	80.3	75
F23	3-methyl-cyclopentanone	86.1	82				
